# Complete chloroplast genome sequence of an endangered *Ottelia cordata* and its phylogenetic analysis

**DOI:** 10.1080/23802359.2020.1768921

**Published:** 2020-05-28

**Authors:** Qingfeng Zhang, Zhixin Shen, Fangyuan Li, Gaojun Li, Jie Shen

**Affiliations:** Hainan Academy of Ocean and Fisheries Sciences, Haikou, China

**Keywords:** *Ottelia cordata*, complete chloroplast genome, phylogenetic analysis, high-throughput sequencing

## Abstract

*Ottelia cordata* is a typical submerged plant to the family of hydrocharitaceae. In this work, complete chloroplast genome of *O. cordata* was assembled and characterized from high-throughput Illumina sequencing data. The chloroplasgenome of *O. cordata*is 157,869bp in length. The *O. cordata* cp genome encodes 128 functional genes, including 85 protein-coding genes, 35 tRNA genes, and 8 rRNA genes. The GC content of the genome was 36,57%. Its nucleotide composition is asymmetric with an overall A + T content of 63.44%. Phylogenetic analysis indicated that *O. cordata* is closely related to the *Elodea.* These results could offer molecular markers and helpful in understanding about the role of *O. cordata* evolution and protection.

## Introduction

*Ottelia cordata* is an annual aquatic herbaceous plant, which belong to the genius *Ottelia Pers* of the family Hydrocharitaceae. It is only distributed in Hainan province in China and also in Myanma, Thailand, and Cambodia (Xu et al. 2010). *Ottelia cordata*was endangered in recent years due to various environmental threats, such as wetland destruction, invasion of alien species and water pollution. In this work, we assembled and characterized *O. cordata* complete cp genome. These results were beneficial for resources study and protection of *O. cordata*.

The fresh *O. cordata* leaves were collected from Zuntan, Haikou (110°17′1.97″E, 19°47′14.43″N). The specimen was deposited at Hainan Academy of Ocean and Fisheries Sciences (P-2019-ZTO1). Total DNA was extracted using Ezup DNA kit and sequenced with Illumina Hiseq 4000. *Ottelia cordata* cp genome sequence was assembled by SPAdes v.3.5.0 and annotated by DOGMA (Wyman et al. 2004). The tRNA was annotated by the method of tRNAscan-SE2.0 (Lowe and Chan 2016) and ARWEN (Laslett and Canback 2008). The size of the *O. cordata* cp genome was 157,869 bp (Genbank accession number: MN056354), which has 10 bp difference with 157,879 bp in Wang’s research (Wang et al., 2019). This genome included a LSC region of 87,672 bp, a SSC region of 19,093 bp, and a pair of IRs (IRa and IRb) of 25,552bp each. Totally, 128 functional genes were identified in *O. cordata*, including 85 protein-coding genes, 35 tRNA genes, and 8 rRNA genes. Totally, 44 SSRs and 43 pairs of large repeat sequences were found in this genome. Among the 44 SSRs, 14 SSRs were located in protein-coding genes (*atpF, rpoC2, rpoC1, accD, clpP, rps8* and *ycf1*). Among these, 43 pairs of long repeat sequences, 18 long repeat sequences were located in protein-coding genes (*psaA, ycf2*).

**Figure 1.**
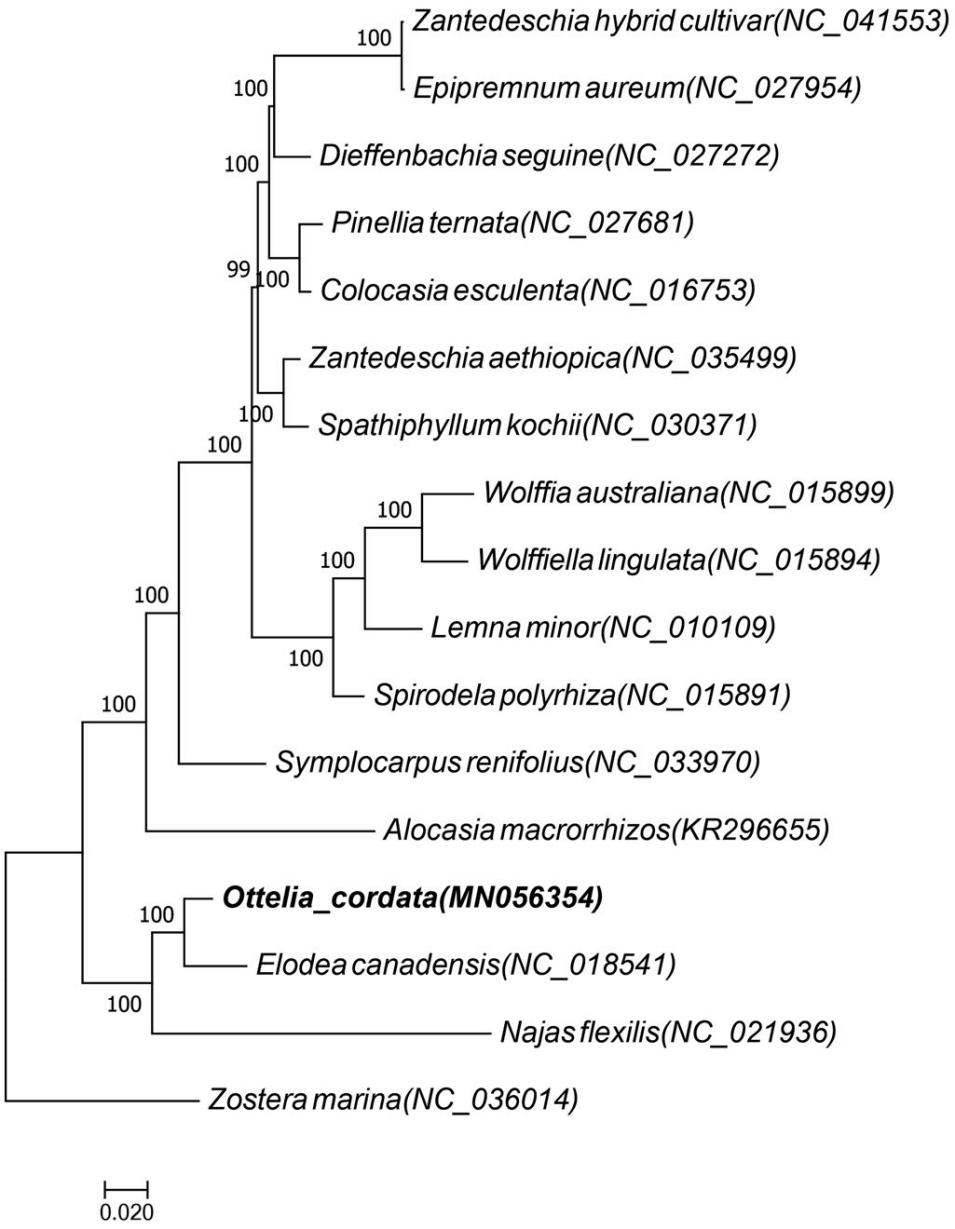
Phylogenetic position of *O. cordata* inferred by ML. The position of *O. cordata* shown in boldface.

In order to ascertain its phylogenetic placement location of *O. cordata,* 17 species respectively belong to three family of *Araceae, Hydrocharitaceae,* and *Zosteraceae* were selected and their common coding genes were analyzed with maximum likelihood (ML) methods by software RAxML8.1.5 with the model of GTR + I+G (Stamatakis, 2014). Among them, 13 species belong to the family of *Araceae* and one species belong to the family of *Zosteraceae.* The result supported the position of *O. cordata* as a sister to Elodea within the Alismatales order (Figure 1). We believe that it will provide new insight into its evolutionary research and conservation.

## Data Availability

The data that support the findings of this study are openly available in figshare at http://doi.org/10.6084/m9.figshare.12089679.
